# United States medical preparedness for nuclear and radiological emergencies

**DOI:** 10.1088/1361-6498/ac0d3f

**Published:** 2021-12-06

**Authors:** Andrea L. DiCarlo, Mary J. Homer, C. Norman Coleman

**Affiliations:** 1 Radiation and Nuclear Countermeasures Program (RNCP), National Institute of Allergy and Infectious Diseases (NIAID), National Institutes of Health (NIH), Rockville, MD, USA; 2 Biomedical Advanced Research and Development Authority (BARDA), Department of Health and Human Services (HHS), Washington, DC, USA; 3 Radiation Research Program (RRP), National Cancer Institute (NCI), National Institutes of Health (NIH), Bethesda, MD, USA

**Keywords:** radiation, preparedness, US Government, biodosimetry, medical countermeasures, animal models, planning, CONOPS, COVID-19, nuclear detonation, radionuclide, decorporation, stockpile

## Abstract

With the end of the Cold War in 1991, U.S. Government (USG) investments in radiation science and medical preparedness were phased out; however, the events of September 11^th^ , which involved a terroristic attack on American soil, led to the re-establishment of funding for both radiation preparedness and development of approaches to address injuries. Similar activities have also been instituted worldwide, as the global threat of a radiological or nuclear incident continues to be a concern. Much of the USG’s efforts to plan for the unthinkable has centered on establishing clear lines of communication between agencies with responsibility for triage and medical response, and external stakeholders. There have also been strong connections made between those parts of the government that establish policies, fund research, oversee regulatory approval, and purchase and stockpile necessary medical supplies. Progress made in advancing preparedness has involved a number of subject matter meetings and tabletop exercises, publication of guidance documents, assessment of available resources, clear establishment of anticipated concepts of operation for multiple radiation and nuclear scenarios, and identification/mobilization of resources. From a scientific perspective, there were clear research gaps that needed to be addressed, which included the need to identify accurate biomarkers and design biodosimetry devices to triage large numbers of civilians, develop decorporation agents that are more amenable for mass casualty use, and advance candidate products to address injuries caused by radiation exposure and thereby improve survival. Central to all these activities was the development of several different animal constructs, since efficacy testing of these approaches requires extensive work in research models that accurately simulate what would be expected in humans. Recent experiences with COVID-19 have provided an opportunity to revisit aspects of radiation preparedness, and leverage those lessons learned to enhance readiness for a possible future radiation public health emergency.

## 1. Introduction

“If you fail to prepare you are preparing to fail” (often ascribed to Benjamin Franklin) is a quote that represents an excellent place to start thinking about medical preparedness for radiological or nuclear public health emergencies. There are several characteristics that distinguish these kinds of incidents from other catastrophes, which include: a) underlying societal radio-phobia and distrust of nuclear power, b) justifiable sense of horror when considering a scenario resulting from a nuclear detonation, c) ease in measuring radiation can produce inappropriate panic (e.g., from the clicking of a Geiger counter on a wrong setting, for what may just be normal background), d) confusion in the units of measure and different systems (e.g., English vs metric), e) political, anti-nuclear agendas, f) body of knowledge not likely to be encountered by emergency responders, and g) potential for misinformation from a variety of news and internet sources.

There are also major differences in terms of preparedness and response among identified radiological and nuclear scenarios. A nuclear powerplant incident, for example, may or may not be contained, which could depend on the reactor construction. If uncontained, it will have local and distant consequences from the plume that includes relatively short-lived radioactive iodine, which is particularly problematic for young children, and longer-lived cesium-137 that can enter the food chain. A radiological dispersal device also has a wide range of scenarios that will require mitigation of internal contamination of a particular radionuclide. A criticality incident can have both serious local destruction and a plume. A nuclear detonation, ranging from an improvised nuclear device to a state-sponsored nuclear weapon, will have an enormous local, regional, and global impact. In this chapter, the authors focus on the consequencse of a nuclear detonation, as the preparedness, planning, response, and recovery for these incidents also include aspects of response that are important for other kinds of scenarios.

An essential feature of a nuclear or radiological incident is that within minutes, there will be a massive public health and medical disaster, with responses needed immediately and the situation rapidly evolving. It is often a considered “low risk-high consequence” event, with various opinions on the type of preparation needed. Some may feel that the risk is so low and the management so unwieldy that, in the face of limited resources for disaster planning, little preparedness is attempted. Unlike the weeks or months of time available to deal with an emerging infectious disease, radiation preparedness must already be in place, and requires extensive training, so that governments, industry, individuals and global partners can respond immediately to an incident.

## 2. Background

In 2005, the Federal Emergency Management Agency (FEMA) prepared 15 National Planning Scenarios (later updated by the Department of Homeland Security (DHS) [1], the first of which was a 10 kiloton (kt) nuclear detonation. These formed the basis for national planning from multiple federal agencies, along with state, local, territorial, and tribal partners. As will be detailed, below extensive preparedness was subsequently undertaken. The radiation sciences community responded rapidly with a major national workshop that included defining “moderate dose” radiation, between low dose environmental considerations and clinical radiation therapy in a publication titled “Molecular and cellular biology of moderate-dose (1–10 Gy) radiation and potential mechanisms of radiation protection: report of a workshop at Bethesda, Maryland, December 17–18, 2001” [2]. The sensitivity of the terrorist situation led to some delay in publication of the report; nonetheless, civilian preparedness and planning began in earnest in 2003.

A unique public document “Planning Guidance for Response to a Nuclear Detonation” [3] was published with input from a wide range of federal agencies in 2009 and was updated in 2010. This guidance provides detailed technical data for planners including the physical damage, casualty types and numbers, medical management, messaging and recovery. Over the past few decades, there have been several national level preparedness exercises conducted, for example, centering on nuclear power plant accidents/attacks and nuclear detonation. Agencies requiring formal preparations for nuclear incidents, such as the Nuclear Regulatory Commission, form a key backbone of U. S. Government (USG) preparedness [4]. The Project BioShield Act, was passed by the US Congress in 2004, with the goal to “accelerate the research, development, purchase, and availability of effective medical countermeasures against biological, chemical, radiological, and nuclear (CBRN) agents” [5]. The act was later updated as the Pandemic and All-Hazards Preparedness and Advancing Innovation Act of 2019 (PAHPAIA), to reauthorize and strengthen HHS efforts to respond to disasters and threats^4^.

This chapter provides an overview of the preparedness actions and tools that have been developed by the USG, with a focus on the public health, medical diagnostics, and medical countermeasures. Notably, an update of the USG’s June 2010 Planning Guidance for Response to a Nuclear Detonation [3] is in preparation and is expected to be released in 2021.

## 3. Preparedness, planning and response - the Nuclear Incident Medical Enterprise (NIME)

**Figure 1.**
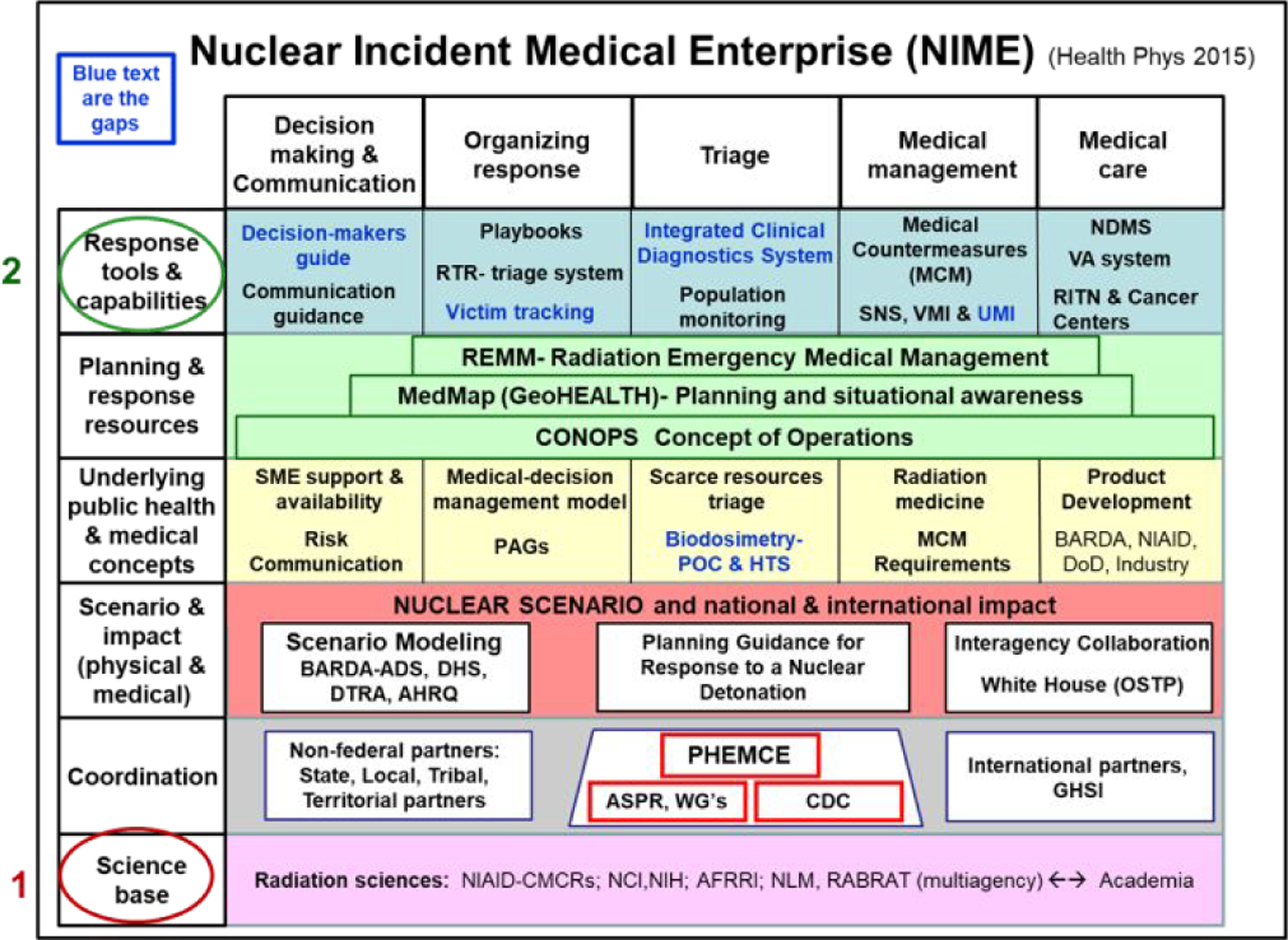
The USG Nuclear Incident Medical Enterprise (NIME) Framework (Used with permission from Wolters Kluwer Health, Inc.) [6]. Figure abbreviations used: Agency for Healthcare Research and Quality (AHRQ); Analytical Decision Support – BARDA (ADS); Armed Forces Radiobiology Research Institute (AFRRI), Office of the Assistant Secretary for Preparedness and Response (ASPR); Biomedical Advanced Research and Development Authority (BARDA); Centers for Disease Control and Prevention (CDC); Concept of Operations (CONOPS); Centers for Medical Countermeasures Against Radiation (CMCRs); Defense Threat Reduction Agency (DTRA); Department of Defense (DoD); Department of Homeland Security (DHS); Global Health Security Initiative (GHSI); High Throughput Screening (HTS); DHHS/ASPR interactive tool (MedMap); Medical Countermeasure (MCM); National Cancer Institute (NCI); National Institute of Allergy and Infectious Diseases (NIAID); National Institutes of Health (NIH); National Disaster Medical System – DHHS (NDMS); National Library of Medicine (NLM); Office of Science and Technology Policy (OSTP); Point of Care (POC); Protective Action Guides - Environmental Protection Agency (EPA) (PAGs); Public Health Emergency Medical Countermeasures Enterprise (PHEMCE); Radiation Bioterrorism Research and Training (RABRAT); Radiation Emergency Medical Management (REMM); Radiation Injury Treatment Network (RITN); Radiation TReatment, TRiage and TRansport system (RTR); Subject Matter Expert (SME); Strategic National Stockpile (SNS), User-Managed Inventory (UMI); Vendor-Managed Inventory (VMI); Veterans Administration (VA); Working Groups (WG).

Compressing a substantial effort by government, academia, industry, and individuals into a single document is not possible; however, highlights are presented to demonstrate the extensive efforts undertaken and the products developed. Figure 1 is an adaptation from a publication on “Public health and medical preparedness for a nuclear detonation: the nuclear incident medical enterprise” [6].

Preparedness needs to be based on the best science (Figure 1, red circle). This approach necessitates a system that can be readily updated. The types of knowledge, processes and capabilities (left hand column) build up to the top row (Figure 1, green circle), which includes available response tools and capabilities. When this approach was developed stepwise starting in 2003–2004, it was recognized that a nuclear incident was likely to be very complicated, yet the primary audience for the information was planners, and in particular, responders. Thus, it is prudent that one keeps in mind that the goal of preparedness and planning is to address the most likely question from a responder: “What do I do now??

Aspects of NIME are next described and information in this Figure 1 is continuously updated. The Science Base (circled in red) depends on federal agencies, academia, professional societies, state and local governments, industry, territorial and tribal partners, and others. Coordination is essential, much of it among subject matter experts who exchange information at annual professional society meetings and international conferences (e.g., Health Physics Society, National Council on Radiation Protection and Measurements, Radiation Research Society, WHO, ConRad, etc.). Scenarios, the next level up in NIME, are essential for planning and conducting exercises. These Planning Guidance documents provide information to planners so that collaboration is facilitated by common knowledge.

For *Underlying Health and Medical Concepts* (Figure 1, yellow rectangles), new ideas needed to be developed. These advances require enhancement of the Science Base, and led to establishment of biodosimetry and medical countermeasure (MCM) funding programs within both the National Institute of Allergy and Infectious Diseases (NIAID) in 2004 and the Biomedical Advanced Research and Development Authority (BARDA) within the Office of the Assistant Secretary for Preparedness and Response (ASPR) in 2006. All the science and basic concepts, such as the Protective Action Guidelines for radiation exposure, now come together in the next level up - Planning Tools and Resources (light blue rectangles). In this level, a response is conceptualized in the Concept of Operations (CONOPS), which includes how it can be planned, orchestrated, modified, and operationalized in as close to real-time as possible. A geographic information system (GIS)-based approach has been developed by the ASPR, now called GeoHEALTH [7]. It was recognized very early on that the complexity of a response will be overwhelming, and that an algorithm approach to information and management would be essential. Perhaps the most unique and valuable tool was created in a partnership between the ASPR and the National Library of Medicine, was a website resource called Radiation Emergency Medical Management ([8]. Critical for this manuscript is that REMM includes references, citations, learning modules, medical order sheets, and more, including many of the documents mentioned here.

The top row of Figure 1 (#2) includes the various tools used in incident management. Medical triage is critical, and while responders have triage systems with which they are familiar, the presence of radiation makes organizing a response difficult. To address that concern, the RTR (Radiation TRiage-TReatment-TRansport) approach was developed that defines spontaneously forming sites (RTR 1–3) and pre-located facilities on GeoHEALTH, including GIS-located assembly, medical, and evacuation centers [9]. To provide surge capacity for management of radiation injury (not burns or trauma) the Radiation Injury Treatment Network (RITN) was established [10]. A Decision-Makers Guide also was produced for government leaders who must manage an incident with a very short time to learn the essentials of a nuclear incident [11].

A new resource was created in 2018 - the “Exposure And Symptom Triage” (EAST) Tool to Assess Radiation Exposure After a Nuclear Detonation [12], “to assist responders in making the initial triage and help direct patients who need immediate medical attention to the proper next station/facility and send those without life-threatening conditions home for now or to care beyond the disaster scene.” An overarching key reason that planning and a well-organized response is necessary is to avoid a crisis standards of care setting, which occurs when there are not sufficient resources for each patient, and some patients who might otherwise survive are triaged to an “expectant” category, until resource balance is attained [13]. A key concept in triage is that patients must be re-triaged when resources do arrive, which could alleviate a crisis standards of care situation.

A new concept proposed in 2019 is that of the Chemical Biological Radiological Nuclear Explosive (CBRNE) Medical Operations Science Support Expert (CMOSSE) [14]. This publication describes a medical competency (not a board or license) for individuals who have both subject matter expertise and disaster operational experience, so that they can work in contact with major decision-makers in guiding a medical response. The aim is to consider implementation of this concept in the next few years.

## 4. Consideration of global preparedness and US role

The spectrum of potential global disaster preparedness, formulated shortly after the September 11, 2001 incident (1), is continuously updated as situations change. These scenarios include newly emerging infectious diseases, nuclear proliferation, and the impact of the changing climate. In 2014, the WHO established the voluntary Joint External Evaluation (JEE) [15] as a component of the International Health Regulations (IHR), which were originally established in 2005. As articulated on the ASPR web site, the JEE is “an international legal framework for global health security, where countries build their own health security capacities and take part of a global surveillance and response network created through the IHR”. The JEE is part of the U.S. Health Security National Action Plan: Strengthening Implementation of the International Health Regulations. “The activities proposed in the plan address capacity gaps found by national and international subject-matter experts during the JEE, and also further the multi-sectoral and multi-disciplinary approach promoted by the IHR to adequately prevent, detect, and respond to public health security threats.” The WHO’s US JEE Mission Report [16] outlines the capacity of the US to respond to “public health threats of a natural, deliberate or accidental nature”. Among several recommendations from the 2016 JEE assessment, the WHO found that the US could “benefit from developing a more formal One Health strategy that encompasses the federal, state and local levels”, highlighted “need for improvements in the national biosafety and biosecurity technical area”, and noted that “it would be beneficial to develop improved collaboration and information sharing across the radiological and health sectors”. The report also encouraged the US to continue “upholding its international roles and participating in relevant international networks…”

There are also plans that have been developed by the WHO to guide radiation preparedness in Europe, as outlined in the Action Plan to Improve Public Health Preparedness and Response in the WHO European Region 2018–2023 [17]. The strategic pillars of this document include plans to build and maintain core capacities, enhance management of events and IHR compliance, track progress, and promote reporting and assessment.

Finally, the North Atlantic Treaty Organization (NATO) and the International Atomic Energy Agency (IAEA) have both played important roles in assessing and ensuring global preparedness for a radiological or nuclear incident. NATO meetings focused on development of clinical tools for triage use [18, 19] have led to important advances in radiation biodosimetry, and tabletop training and educational exercises [20–22] have identified critical aspects of radiation medical response. IAEA’s many retrospective analyses of radiation incidents [23–27] have help to identify best practices, and provide lessons learned from radiation incidents all over the world.

## 5. Initiating and advancing medical preparedness – scenarios of concern and areas of scientific interest

Since early 2004, when the USG began in earnest to implement programs to address significant gaps in medical preparedness for a radiological or nuclear incident, activities have focused on funding research on radiation medical countermeasures (MCMs), biomarker discovery, and biodosimetry device development, to advance products toward licensure, purchase, and stockpiling [28, 29]. Early stage research has centered on animal model development, with the goal being to test MCMs for mitigation/treatment of the acute radiation syndrome (ARS) and/or the delayed effects of acute radiation exposure (DEARE) [30]. Affected organ systems from radiation exposure include the hematopoietic [31, 32], gastrointestinal (GI) [33], cutaneous [34], pulmonary [35–37], renal [38], cardiovascular [39] and/or central nervous system [40] compartments of the body. Radiation combined injuries (radiation plus trauma, such as burn or blast wounds) are also a concern [41]. In order to effectively address these kinds of biological damage, it is essential to triage potentially large numbers of affected persons, based on the level of radiation exposure that a patient may have received (biodosimetry), and the potential health impacts of that exposure to the different organ systems (biomarkers and organ-specific, predictive biodosimetry). Within triage, the focus is to distinguish between concerned citizens who received little or no radiation exposure, and therefore, are not in need of care, from those that would benefit from provision of some form of medical management.

Following guidance and provision of funding from multiple agencies (e.g., NIAID, BARDA, the Food and Drug Administration (FDA); and the Department of Defense (DoD)) the USG supports research ranging from early-stage through advanced development, licensure, and stockpiling of MCMs and biodosimetry products to diagnose, and treat injuries resulting from radiation exposure. Research areas with the greatest programmatic priority include:

1. MCMs that are:
  - Effective to improve survival or reduce morbidities when given a minimum of 24 hours (preferably later) after radiation exposure (the minimum time anticipated for USG responders to identify patients in need and deliver MCMs)
  - Safe and easy to administer to civilians, including special populations (e.g., children, pregnant women, the elderly, and those with underlying diseases)
  - Route of administration amenable to a mass casualty radiation incident based on the timing of when the product would be administered
  - Preferably have broad activity (e.g., potential for use in more than one treatment scenario) and long shelf life e.g., (room temperature preferred to cold storage)
2. Biodosimetry methods or devices that are:
  - Minimally invasive (e.g., finger-sticks are preferred instead of venipuncture)
  - Capable of measuring absorbed radiation dose from internal and/or external radiation exposure
  - Able to rapidly and accurately distinguish people who need treatment from those who do not for either field use or a definitive care setting, such as a hospital or trauma center
3. Drugs to remove or block radioactive materials from being incorporated into tissues :
  - Effective when administered at 24 hours or later after internalization of a radionuclide
  - Ideally able to remove more than one type of radionuclide from the body
  - Blocking radionclide uptake into sensitive organs by physical means (e.g., inducing coughing to remove inhaled particles in the lung via mucociliary clearance, or preventing thyroid accumulation of radioactive iodine), or binding to radionuclides in the blood, GI tract or another organ to expedite their removal
  - Improvement of existing agents, by modifying them to be more amenable for mass casualty use (e.g., going from an intravenous (IV) to an oral or subcutaneous (SC) formulation

## 6. Concept of Operations (CONOPS)

Specific to a radiation mass casualty incident, several factors must be considered early during the development of MCM and biodosimetry approaches, to ensure maximum usability of any product for a public health emergency. Emergency response to a mass casualty radiological/nuclear event will likely follow two general phases of treatment: field care and definitive care [42]. The field (or pre-hospital) care phase generally includes the first 72 hours of the emergency response. During this time, resources and trained personnel are expected to be exceedingly scarce, thus delaying and limiting access to advanced medical care and treatments. Because there will be a lag time between any incident and the USG’s ability to access the site, assess the nature of the injuries sustained, and mobilize resources, it is generally understood that few drugs would be available within the first 24 hours after the incident. In addition, given the uncertainty of patient monitoring, drugs that require only one or a few treatments would be prioritized. Product formulations for civilian populations that can be easily administered in a mass casualty scenario (e.g. via oral, SC, trans-cutaneous, intramuscular or inhalation routes) are also desirable. An agent with dual utility, for routine patient care and emergency management, is preferable, in that there will be a local supply already on site and medical care providers will have familiarity with its use. Although it may be possible for IV treatments to be administered, these routes would be more difficult to provide in a field-setting, and might be best considered as a definitive care treatment. The definitive care phase is administered at medical centers or hospitals and generally extends beyond the initial 72 hours of the emergency response. Definitive care includes the full range of medical support and treatments necessary to manage a patient’s condition.

### 6.1 Treatment provided with incomplete biodosimetry

Because it unlikely that healthcare providers will know precisely (if at all) the amount of absorbed radiation for each patient, nor will they be aware of any shielding of the body, MCMs must exceedingly safe with a high therapeutic index. Because people who were not exposed to radiation might still receive treatment, drug safety profiles must be strong [43].

### 6.2 Special populations

MCMs are normally first studied in adult animal models of radiation injury (both males and females) [44]; however, it is critical that products are appropriate for all ages and pre-existing conditions and that biodosimetry methods take into account variations in responses across key demographics (e.g., pediatric [45], geriatric [46], pregnant, immunocompromised and with underlying disease.)

### 6.3 Nature and complications of expected casualties

Because a radiological or nuclear incident could involve explosions and fire, it is also important to consider models of radiation combined injuries (RCI), as well as testing the efficacy of MCMs in those models [47]. In addition, many casualties may have only received a partial-body exposure, so these type of radiation models should be considered. Finally, given the 2021 US FDA approval of Nplate® for hematopoietic ARS, and previous approvals of Neupogen® and Neulasta® (2015), and Leukine® (2018), it is important to understand any drug-drug interactions as well as any potential efficacy synergies if multiple products are used [48]. A thorough knowledge of how the products may interact with one another will be essential to ensure careful and appropriate resource management and could be critical in avoiding scarce resource scenarios.

### 6.4 Shelf life and storage conditions

Due to the expense and complication involved in stockpiling an MCM, long shelf-lives are preferred, as is room-temperature storage. Although refrigerator, freezer and liquid nitrogen storage may be possible, these are not desirable, given the potential for limited access to electricity and the challenges associated with stockpiling. USG agencies involved in MCM procurement are also implementing vendor-managed inventory programs for commercial drugs with other indications [14].

### 6.5 Stockpiling strategies and repurposing

The USG has employed several strategies to stockpile MCM inventories. For “buy and hold” actions, the Strategic National Stockpile (SNS) takes control of the product and stores it at SNS sites. The product is discarded when it reaches expiry. The SNS has introduced shelf-life extension programs (SLEP) to enable extension of the expiry, in order to improve the return on investment of the USG’s purchase. Some radiological/nuclear MCMs are held in USG buy and hold programs, as there are limited customers for their market outside of the government.

In the procurement of MCMs, the USG has the potential to look for products in the commercial market, which has many advantages including familiarity of use for the end users and medical community, the potential for a more accelerated path to MCM indication approval, and significant savings for the taxpayer in both development and procurement costs. The radiological and nuclear space has the potential to identify analogous injuries being addressed in the commercial market, that will allow the USG to leverage off of these markets for MCM preparedness, through either a vendor- or user-managed inventory (VMI or UMI). Commonly referred to as repurposing [49], this approach has great potential for building comprehensive preparedness, and saving time and resources by leveraging synergies between compatible indications; i.e., indications in development with the same formulations and dosing can use the same preclinical, safety, and CMC activities to support approval for both indications. Likewise, already approved commercial products can be leveraged in a similar way if the proposed MCM has the same formulation and dosing regimen as the product in the market. Some of the markets that have been leveraged include oncology, conditions caused by inflammatory cascades, immune cascades, apoptotic cascades, etc. Vendor managed inventories have the product stored in the vendor’s warehouse and stock is rotated to avoid expiry [50].

Optimally, it would be advantageous to identify large enough markets where the requirement could be completely covered with existing inventory, but the USG can also (e.g., myeloid cytokines) support a vendor managed inventory bubble. This strategy also has the advantage of making long-term stability concerns irrelevant. In UMI (also referred to as forward-deployment), the product is stored at the end-user sites such as hospitals, pharmacies, and emergency vehicles [51]. This strategy is key for products that require rapid administration after injury, and an “inventory bubble” of stock is rotated through ongoing use, which avoids the problem of drug expiry.

## 7. Regulatory considerations

Translational research for the development of MCMs can be accelerated by addressing regulatory challenges proactively. The US FDA has developed published guidances that provide a pathway for sponsors seeking approval/licensure/clearance of approaches to address radiation mass casualty care [52, 53]. The FDA’s regulations concerning the approval of new drugs or biological products when human efficacy studies are neither ethical nor feasible are known as the Animal Rule (21 CFR 314.600 for drugs; 21 CFR 601.90 for biological products). This path to FDA approval is applicable to MCMs, and it is vitally important that groups involved in MCM development understand these regulations. The Animal Rule states that FDA will rely on evidence from animal studies to provide substantial evidence of effectiveness only when these criteria are met:

- Reasonably well-understood pathophysiological mechanism of the toxicity of the substance and its prevention or substantial reduction by the product
- Effect is demonstrated in more than one animal species expected to react with a response predictive for humans, unless shown in a single animal species that represents a sufficiently well-characterized animal model for predicting the response in humans
- Animal study endpoint is clearly related to the desired benefit in humans, generally the enhancement of survival or prevention of major morbidity
- Data or information on the kinetics and pharmacodynamics of the product or other relevant data or information, in animals and humans, allows selection of an effective dose in humans

## 8. Animal model development and MCMs

To address these US regulatory requirements, government agencies have supported development of both small and large animal models for a number of different manifestations of radiation injury [44, 54]. These representative creatures have been developed based on a basic understanding of the mechanisms of radiation injury, and have helped in the identification of pathways that could be targeted for MCM development, as well as providing models that simulate anticipated human responses to radiation exposure. Generally, rodents, including mice, rats, and slightly larger animals like rabbits and guinea pigs, are appropriate small animals, and are especially useful for early MCM development. They are frequently used to work out dosing, dose regimens, formulation, biomarker identification, and route of administration. Once these factors have been determined, successful products and/or biomarkers are explored in large animals, such as nonhuman primates (NHPs), mini, and full-size pigs. The selection of the most appropriate animal model is normally dependent on the target of the MCM or biodosimetry marker. For example, whereas NHPs are believed to be the best model to approximate ARS and DEARE syndromes like hematopoietic injury, GI damage and late lung complications, swine models are better to evaluate skin-focused biomarkers, injuries and MCMs.

There are also several multi-organ, systemic impacts of radiation injury where vascular injury is likely a major component of the etiology. Assessment of these effects, such as radiation-induced coagulopathies, endotheliopathies and other broad damage can also be assessed in models like rabbits, whose clotting and vessel injury responses are like humans [55]. To establish efficacy of an MCM, clinically relevant improvements in outcomes like survival and/or a lessening in the severity of other injury endpoints (e.g., radiation-induced pneumonitis or fibrosis) should be assessed and the product should provide convincing evidence of a health benefit.

## 9. Biomarkers of radiation injuries and biodosimetry

As of this writing, no biodosimetry approaches have yet been cleared by the US FDA for use in radiation mass casualty triage. USG investments in biomarker discovery and device design spans exploratory studies into novel markers, through funding of more standard cytogenetic and “omics” technologies, to advanced development of prototypes and validation of biomarker panels [56]. In advanced development, there are many challenges involved in identifying laboratories and instrumentation and ensuring safe transport of samples and reagents [42, 56]. Testing should also involve little training with robust and accurate responses. Coupled with other clinically focused injury assessments such as MEdical TREatment ProtocOLs for Radiation Accident Victims (METREPOL) [57], these panels of markers and devices can provide a more complete clinical picture to guide patient care. As has been seen with COVID-19 testing, these hurdles are not only important for assessment of radiation injury. Discovery of early biomarkers that can predict late injuries, as well as development of point-of-care, field deployable tests, and approaches that can help guide physician actions in definitive care setting are all critical to providing timely and necessary medical care.

## 10. Removal/blocking of radionuclides

The last focus areas to ensure radiation medical preparedness is support of studies on products to remove (decorporate) or block the effect of internalized radionuclides [58]. Although several products are licensed (e.g., Ca- and Zn-DTPA, Prussian blue, and potassium iodide) aspects of their effective use in a mass casualty setting are impacted by factors such as route of administration, or limited radionuclide targeting. For these reasons, approaches that modify formulations of existing drugs to make them orally available [59–61], expand the range of accessible radionuclides [62], or serve to reduce radiation body burden through approaches such as mucociliary clearance [63–66] remain areas of research interest.

## 11. Lessons learned

Preparedness planning is an iterative process and as additional information and new gaps are identified; they can be addressed through the schema presented earlier. Training exercises (such as Gotham Shield 2017) [67], and response efforts for emerging infectious diseases such as COVID-19 provide valuable lessons and new information for consideration. What has been made very clear from both exercises and actual response efforts is that effective communication is paramount at all levels, between response organizations, between administrative leadership and operational response units, and with the public. Moreover, it is also clear that there is always room for improvement. Additionally, the critical role of the CMOSSE and what can happen with insufficient expertise informing decisions has also been made clear through exercises and response operations [14]. Planning efforts moving forward should be clear about the role of the CMOSSE and where their input should help inform decision making. An additional area for improvement should involve the review of response processes with the aim of reducing administrative burden and unnecessary steps to improve efficiency and speed of actions.

Finally, from a scientific research focus perspective, it is clear from the etiology of COVID-19 that our ability to treat certain pathophysiologies is impaired by insufficient natural history along the course of disease and the lack of therapeutics or clinical management approaches to address COVID-19 patients at these different time points along the continuum of care. Particularly, endotheliopathy and coagulopathy conditions, along with the inflammatory response seen in COVID-19 patients, have posed challenges for patient diagnosis, treatment and management, not only during active infection but, also after patients have recovered from the acute phase of disease but are still experiencing long-lasting sequelae. The systemic nature of SARS-CoV2 in infecting endothelial cells is not unlike the systemic injury to the endothelial system seen with acute exposure to ionizing radiation and therefore parallel threat-agnostic approaches can be leveraged and considered for patient management [68]. There are several areas of the USG radiological/ nuclear countermeasures programs that could be leveraged to improve our knowledge in these areas, develop new targets for MCM development, and test existing MCMs in the radiation portfolio for their ability to treat COVID-19 and future emerging infectious diseases, and restore homeostasis.

## Footnotes

4
https://www.congress.gov/bill/116th-congress/senate-bill/1379/text
